# Disulfide isomerase-like protein AtPDIL1–2 is a good candidate for trichlorophenol phytodetoxification

**DOI:** 10.1038/srep40130

**Published:** 2017-01-06

**Authors:** Ri-He Peng, Jin Qiu, Yong-Sheng Tian, Jian-jie Gao, Hong-juan Han, Xiao-Yan Fu, Bo Zhu, Jing Xu, Bo Wang, Zhen-jun Li, Li-juan Wang, Quan-Hong Yao

**Affiliations:** 1 Agro-Biotechnology Research Institute, Shanghai Academy of Agricultural Sciences; Shanghai Key Laboratory of Agricultural Genetics and Breeding, 2901 Beidi Rd., Shanghai, People’s Republic of China

## Abstract

Trichlorophenol (TCP) is a widely used and persistent environmentally toxic compound that poses a carcinogenic risk to humans. Phytoremediation is a proficient cleanup technology for organic pollutants. In this study, we found that the disulfide isomerase-like protein AtPDIL1–2 in plants is a good candidate for enhancing 2,4,6-TCP phytoremediation. The expression of *AtPDIL1*-*2* in *Arabidopsis* was induced by 2,4,6-TCP. The heterologously expressed AtPDIL1-2 in *Escherichia coli* exhibited both oxidase and isomerase activities as protein disulfide isomerase and improved bacteria tolerance to 2,4,6-TCP. Further research revealed that transgenic tobacco overexpressing AtPDIL1-2 was more tolerant to high concentrations of 2,4,6-TCP and removed the toxic compound at far greater rates than the control plants. To elucidate the mechanism of action of AtPDIL1-2, we investigated the chemical interaction of AtPDIL1-2 with 2,4,6-TCP for the first time. HPLC analysis implied that AtPDIL1-2 exerts a TCP-binding activity. A suitable configuration of AtPDIL1-2-TCP binding was obtained by molecular docking studies using the AutoDock program. It predicted that the TCP binding site is located in the b-b′ domain of AtPDIL1-2 and that His254 of the protein is critical for the binding interaction. These findings imply that AtPDIL1-2 can be used for TCP detoxification by the way of overexpression in plants.

Given their broad-spectrum antimicrobial properties, chlorinated phenols (CPs) have been used as components for the preservation of wood, paints, vegetable fibers, and leather, as well as for the synthesis of intermediates in the manufacture of herbicides, fungicides, pesticides, insecticides, pharmaceuticals and disinfectants^1^,^2^,^3^. Trichlorophenol (TCP) is an important kind of CPs and often considered as biomarker of many organochlorinated compounds^4^. TCPs are abundant in environments exposed to chemical industry effluents or heavy pesticide usage^5^. They are difficult to remove from the environment because of their numerous origins and are barely biodegradable; hence, humans are exposed to TCPs through diet, water and air^6^. At present, TCP pollution threatens the safety of human health because of its carcinogenic, mutagenic, teratogenic, endocrine disruptors and other characteristics^7^,^8^,^9^.

The need to restore TCP-contaminated sites has aroused the development of effective methods for TCP removal in the last few years. Phytoremediation is a simple method of removing, containing, or rendering harmless environmental contaminants^10^. Many mechanisms in the phytoremediation environment may promote the removal of organic contaminants^11^. To date, only a few cases of TCP phytoremediation have been reported. The overexpression of a cotton-derived laccase gene in *Arabidopsis thaliana* metabolizes TCPs *ex situ*, thereby creating a less toxic environment for plant growth^12^. *Arabidopsis* overexpressing *Populus* UGT (Genbank XP_002320190) exhibits a strikingly higher capacity for the phytoremoval and degradation of TCPs by phase II metabolism^13^. Exposure of plants to TCPs causes stress and toxicity, which lead to the poor phytoremediation of TCPs. Low TCP concentrations decrease the chlorophyll fluorescence and biomass accumulation of *Spirodela punctata*, whereas high TCP concentrations increase the severity of the stress^14^. The presence of 2,4,6-TCP greatly reduces the germination capability and inhibits the root elongation of *Arabidopsis* seeds. Gene ontology (GO) analysis showed that TCP-responsive genes are involved in various biological processes, including secondary metabolism and biological regulation related to growth and development^15^.

Protein disulfide isomerase (PDI), a member of the thioredoxin superfamily, is involved in the progression and maturation of secretory proteins in the endoplasmic reticulum (ER)^16^,^17^. PDI catalyzes disulfide bond formation (reduction within the ER or isomerization) and acts as a molecular chaperone in assisting polypeptide folding^18^. Several PDI genes have been identified in different plants^19^. The multiplicity and structure difference of PDI genes in plants suggest that they serve both specialized and overlapping functions to adapt to new biochemical needs or environments. A protein disulfide isomerase-like protein in *Medicago truncatula* is found to be tightly associated with both shoot biomass and leaf size under dehydration stress^20^. PDI is a component of unfolded protein response that alleviates ER stress and lessens programmed cell death^21^. Abolishing the expression of the PDI protein PDIL1-1 induces ER stress and leads to the formation of a floury endosperm in rice, caused by the loose packing of starch granules^22^. Microarray analysis confirmed that the expression of PDIs containing putative transmembrane domains is affected by ER stress responses^23^. Aside from acting as a protein folding catalyst, PDI also serves as an intracellular binding protein for small molecules that contain a phenolic structure, including endogenous hormones (e.g., estrogens and thyroid hormones)^24^,^25^ and environmental chemicals (e.g., Bisphenol A, BPA)^26^.

As a typical phenolic compound, TCP might be a target of PDI, and its binding to PDI might be mechanistically responsible for its adverse effects on plants. Resistance to TCP can be achieved by overexpressing the target protein in plants, and the absorbed TCP can be fixed on the ER and further metabolized. In the present study, we monitored the expression patterns of all PDI genes under TCP stress by using gene chip assay to search for the PDI suitable for TCP binding in plants. Some TCP upregulated genes that encode protein disulfide isomerases were selected from the 2,4,6-TCP-treated *Arabidopsis* gene chip. Direct binding study revealed that the protein AtPDIL1-2 was a target of TCP. The phenotype of AtPDIL1-2 gene transgenic plants supplemented with TCP was evaluated to establish the relationship between TCP detoxification and the protein. We propose that the mechanism of PDI binding to TCP can be used to breed plants for remediating TCP pollution in soil.

## Results

### *ATPDIL1*-*2* is specifically upregulated by 2,4,6-TCP

In *Arabidopsis*, the PDIL clade includes the PDI group, the adenosine 5′-phosphosulfate reductase-like group, and the quiescin-sulfhydryl oxidase group. The upregulated PDIL genes in 2,4,6-TCP-treated plants were searched using cDNA microarray to investigate the possible molecular mechanisms of 2,4,6-TCP in plant ER stress. Quantitative analysis of microarray data identified 13 upregulated PDIL genes between the treated and untreated samples, with P = 0.05 as a cutoff. Among the 13 PDIL genes, *AtPDIL1*-*2* was upregulated with a fold change of 2.39 (Supplementary Table S1).

RT-PCR results revealed that the induction of *AtPDIL1*-*2* was more significant than that of its close homolog *AtPDIL1*-*1* in the presence of 2,4,6-TCP. The effect was more notable at 10 μM than at 30 μM treatment concentration (Supplementary Fig. S1).

### *ATPDIL1*-*2* overexpression improves the resistance of *Escherichia coli* to 2,4,6-TCP

**Figure 1:**
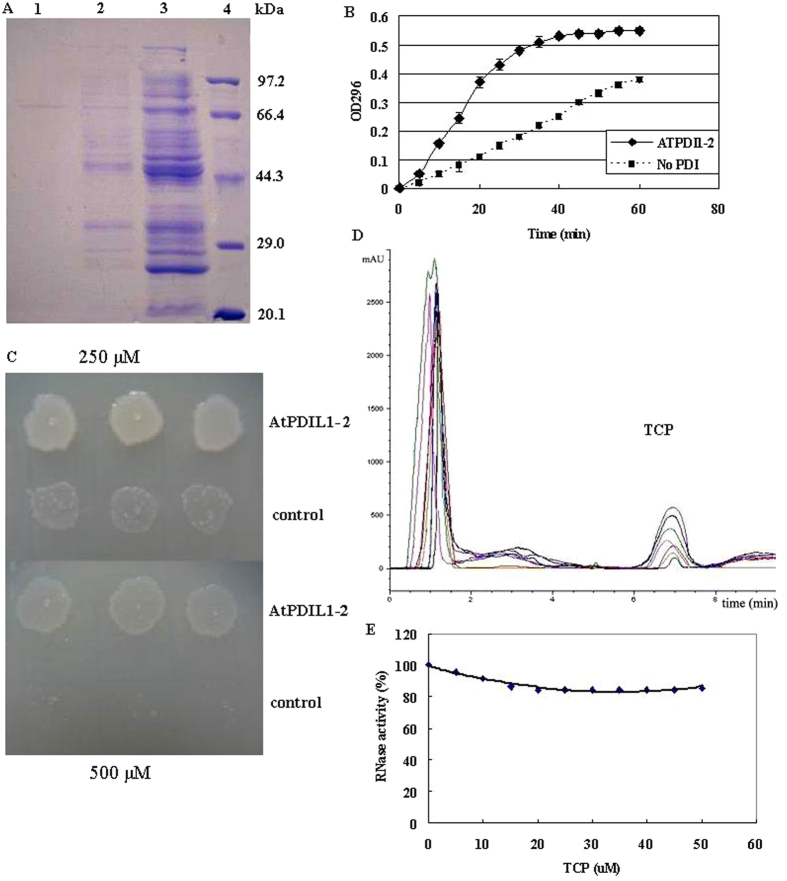
Assay of binding activity of AtPDIL1-2 with 2,4,6-TCP. (**A**) Expression and purification of the recombinant AtPDIL1-2 in *E. coli*. The fractions in purification were applied to a 10% SDS-PAGE gel. lane 1, fraction eluted by 250 mm imidazole and 0.1% sucrose monolaurate; lane 2, washing fraction; lane 3, total induced protein; lane 4, molecular mass standards. (**B**) AtPDIL1-2 catalyzed oxidative folding of reduced denatured RNase on the basis of cCMP hydrolysis, creating a change in the absorbance at 296 nm. (**C**) *E. coli* cells that overexpressed the product of AtPDIL1-2 exhibited significant resistance to 2,4,6-TCP. The cells were diluted to 10^3^ cells/μL, and 0.5–10 μL cells were added to the LB plate supplemented with 250 and 500 μM 2,4,6-TCP. (**D**) Examination of the binding activity of AtPDIL1-2 with 2,4,6-TCP, recombinant AtPDIL1-2 (3.5 μM) was incubated with 50 μM 2,4,6-TCP in 0.5 mL binding buffer [50 mM Tris-HCl buffer (pH 7.0) containing 150 mM NaCl] for 0–3 h at 4 °C. The residual TCP was detected with HPLC with UV detection at 210 nm per 30 min. The x axe was time (minute) and y was mAU (milliabsorbance unit). (**E**) Effects of TCP on PDI-mediated isomerase activity.

The *ATPDIL1*-*2* gene, as a 1527 bp fragment, was amplified and cloned into the expression vector pET-32a to yield plasmid pET-PDIL. Upon induction with isopropyl β-d-thiogalactoside and the *E. coli* strain DH5a (pET-PDIL), ATPDIL1-2 was purified by affinity chromatography. Analysis of the band of the purified protein showed that the recombinant protein was about 72 kDa, corresponding to the calculated molecular masses of the His-tagged ATPDIL1-2 fusion protein (Fig. 1A).

The activity of ATPDIL1-2 in the catalytic oxidative refolding of reduced denatured RNase A was tested; this assay depends on both the oxidase and isomerase activities of PDI. In a spectrophotometric assay for cCMP hydrolysis, RNase activity increased upon the addition of ATPDIL1-2 (Fig. 1B). These results indicated that ATPDIL1-2 can provide the oxidizing equivalents necessary for the oxidative refolding of reduced inactive RNase.

Data showed that 2,4,6-TCP was highly toxic to bacteria. The growth of *E. coli* was inhibited by 500 μM 2,4,6-TCP. However, *E. coli* overexpressing ATPDIL1-2 exhibited less growth inhibition in the presence of the same toxin concentrations (Fig. 1C). This result indicated that *in*-*vivo* AtPDIL1-2 improved *E. coli* resistance to TCP.

### TCP-binding properties of recombinant AtPDIL1-2

The TCP-binding activity of AtPDIL1-2 was characterized using the purified recombinant protein from *E. coli*. When the purified AtPDIL1-2 was added to the binding buffer containing 2,4,6-TCP, the residual TCP was calculated with HPLC based on its standard. The residual TCP in the mix buffer reduced quickly. After 3 h, only a small amount of 2,4,6-TCP can be detected (Fig. 1D). The result confirmed that AtPDIL1-2 possessed 2,4,6-TCP-binding activity. In addition, our experiments showed that TCP can inhibit PDI-mediated RNase refolding activity. The percentage of maximum inhibition by TCP was calculated to be 16% (Fig. 1E). This result suggested that the binding of 2,4,6-TCP to AtPDIL1-2 seriously affected the protein function.

### Screening of the TCP binding site structure of ATPDIL1-2

**Figure 2:**
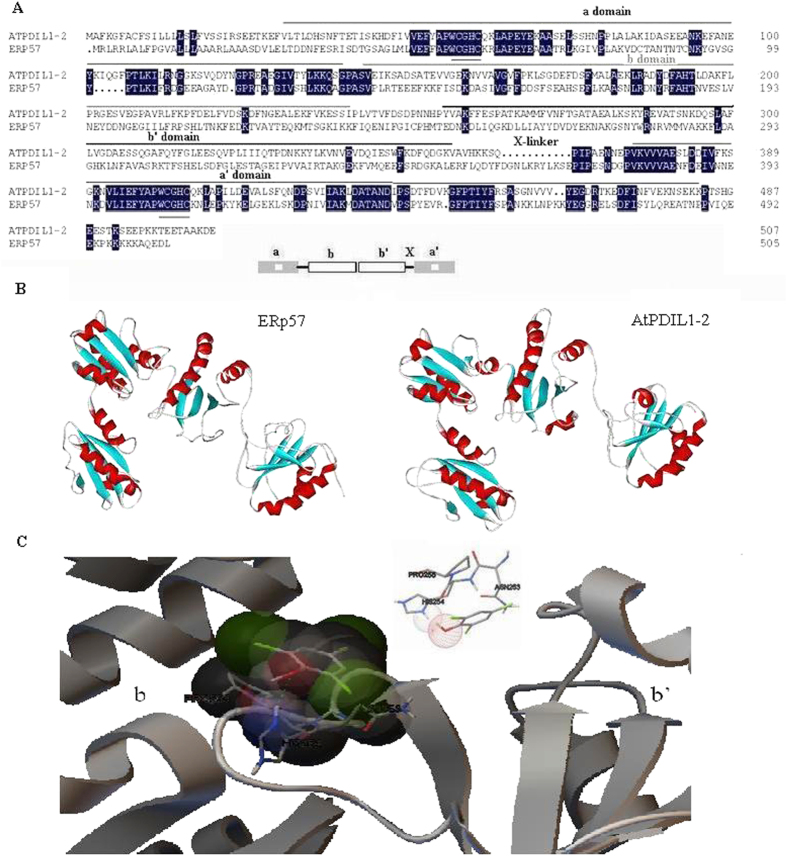
Relative binding activity of AtPDIL1-2 for 2,4,6-TCP. (**A**) Sequence alignment of AtPDIL1-2 and ERp57. Both proteins shared more similarity sequence within the a-type domains than the b-type domains. The regions for oxidoreductase activity were underlined. (**B**) Contrasting the crystal structures of AtPDIL1-2 with ERp57. Both proteins showed similar structures of both a-type and b-type domains. (**C**) Docking analysis of the binding interaction of 2,4,6-TCP inside AtPDIL1-2 with AutoDock. A close-up of the docking result of the AtPDIL1-2–2,4,6-TCP binding mode, showing that a hydrogen bond formed between the 1-hydroxyl group of TCP and His-254 of AtPDIL1-2. Atoms were labeled with different colors. Red: oxygen; white: hydrogen; green: chlorine; gray: carbon. The radius of the sphere represents interaction strength.

Structure analysis showed that AtPDIL1-2 contains two redox-active a-type thioredoxin domains (a, a′) and two redox-inactive b-type domains (b, b′) as the classical PDI ERp57. They were arranged with the architecture a-b-b′-a′. A small linker region X was located between b′ and a′. Both a-type domains that were responsible for the oxidoreductase activity of AtPDIL1-2 have a WCHC active site (Fig. 2A and B).

In addition to peptides and proteins, PDI can bind a diverse set of ligands, such as 17β-estradiol (E2), 3,3′,5-triodo-l-thyronine (T3), and BPA. Equilibrium analysis using rat PDI showed a single E2 binding site. Further research on various human PDI protein fragments showed that its E2-binding activity was associated with the b-b′ domain combination. We hypothesized that AtPDIL1-2 has a similar binding site with 2,4,6-TCP to human PDI for E2-binding. We selected the similar region of AtPDIL1-2 for docking analysis to probe the TCP-binding site by using AutoDock software 4.2.6. Results revealed that 2,4,6-TCP exhibited fairly good docking results into AtPDIL1-2. The ligand was docked deeply within the binding pocket region of AtPDIL1-2 forming hydrogen bonds with His254, and the amino acid residues surrounding the binding pocket (Fig. 2C). The docked TCP exhibited a low RMSD of 0.05 Å, and the binding free energy was −3.32 kcal/mol.

### Overexpression of AtPDIL1-2 enhanced tolerances to TCP in tobacco plants

**Figure 3:**
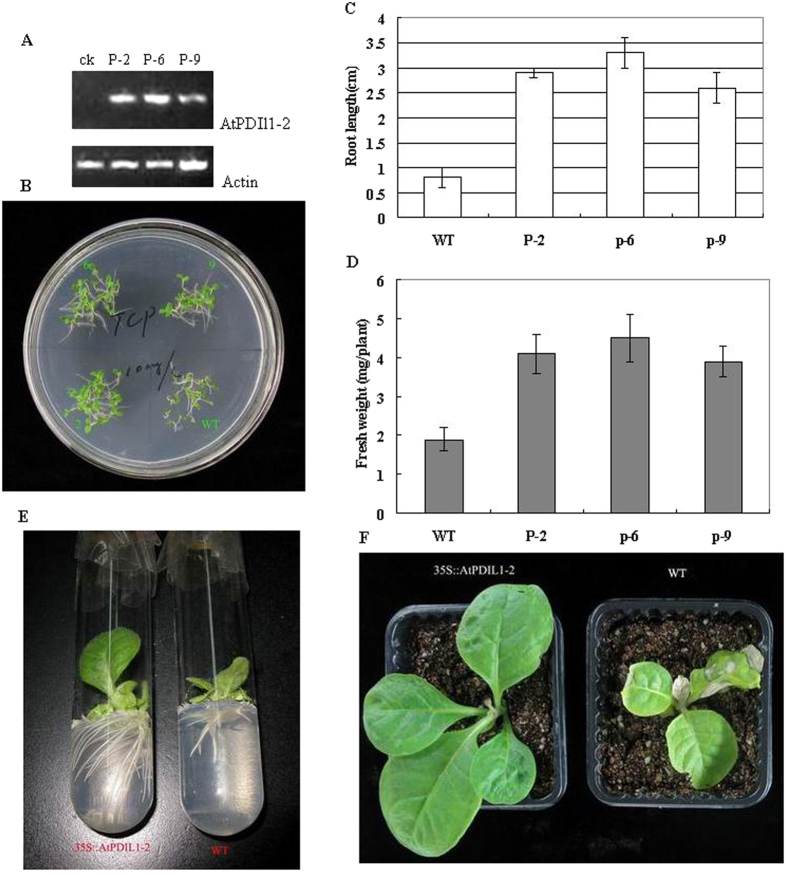
*AtPDIL1*-*2* overexpression enhanced plant tolerance to 2,4,6-TCP. (**A**) RT-PCR analysis of *AtPDIL1*-*2* in 10-day-old seedlings of tobacco transferred with and without *AtPDIL1*-*2*. (**B**) Comparison of transgenic plants and wild type (WT) plants after germinated and grown for 10 days on MS agar plates containing 10 mg/L 2,4,6-TCP. P-2, P-6, and P-9 represent different *AtPDIL1*-*2* transgenic line. (**C**) Comparison of root length of transgenic plants and WT grown on 1/2MS agar plates containing 10 mg/L 2,4,6-TCP for 10 days. (**D**) Comparison of shoot fresh weight of transgenic plants and WT grown on 1/2MS agar plates containing 10 mg/L 2,4,6-TCP for 10 days. (**E**) Tobacco was constantly grown on TCP for 4 weeks. (**F**) TCP treated 4 weeks after the second spraying.

The transgenic lines expressing the *AtPDIL1*-*2* gene were selected from the T2 plants by PCR using specific primers. A RT-PCR product of approximately 260 bp corresponding to the 3′ domain of the coding sequence was detected in three transgenic plant lines (P-2, P-6, and P-9), whereas no fragment was amplified in wild-type (WT) plants. Relatively higher expression levels were detected in P-6 than in the other plant lines (Fig. 3A).

We previously showed that 2,4,6-TCP inhibits the root elongation of *Arabidopsis* seedlings^15^. To investigate whether AtPDIL1-2 is resistant to TCP, the growth of transgenic plants stably expressing *AtPDIL1*-*2* was examined on agar plates containing 10 mg/L 2,4,6-TCP. After 10 days of growth, the *AtPDIL1*-*2* transgenic plants showed higher tolerance to 2,4,6-TCP than the WT plants, as evidenced by the broader leaves and longer roots of the transgenic plants compared with the WT plants (Fig. 3B). The average root length and fresh weight of the *AtPDIL1*-*2* transgenic plants were about 3.3–4.5 and 2.0–2.4 times those of the WT plants, respectively (Fig. 3C and D). The resistance of the transgenic tobacco to TCP was associated with the expression level of AtPDIL1-2. Among the three transgenic lines, P-6 exhibited the greatest seedling weight and the longest root length.

Efficient root formation is a crucial characteristic for plants designed for potential application in phytoremediation. To investigate the root development, the *AtPDIL1*-*2* transgenic line P-6 and WT were transferred to a grass tube with 10 mg/L TCP. After a month of growth, the roots of the *AtPDIL1*-*2* plants developed better than those of the WT plants, as evidenced by the 4.2 times more secondary roots and 3.5 times longer roots in the transgenic plants than the WT plants (Fig. 3E).

Given its COOH-terminal KDEL motif, AtPDIL1-2 is mainly localized in the ER. TCP was stabilized by AtPDIL1-2 overexpressed in the ER, the oxidizing environment that favors more degradation. This phenomenon caused the *AtPDIL1*-*2* transgenic plants to grow in a less toxic or nontoxic microenvironment. A pot soil assay under greenhouse conditions showed that the *AtPDIL1*-*2* plants were generally more resistant to this TCP than the WT plants. A month later, the *AtPDIL1*-*2* P-6 plants produced a biomass that is significantly greater than that of the WT plants. In addition, the WT seedlings exhibited widespread chlorosis after being sprayed with TCP (Fig. 3F).

### Phytoremediation of TCP by Transgenic Plants

**Figure 4:**
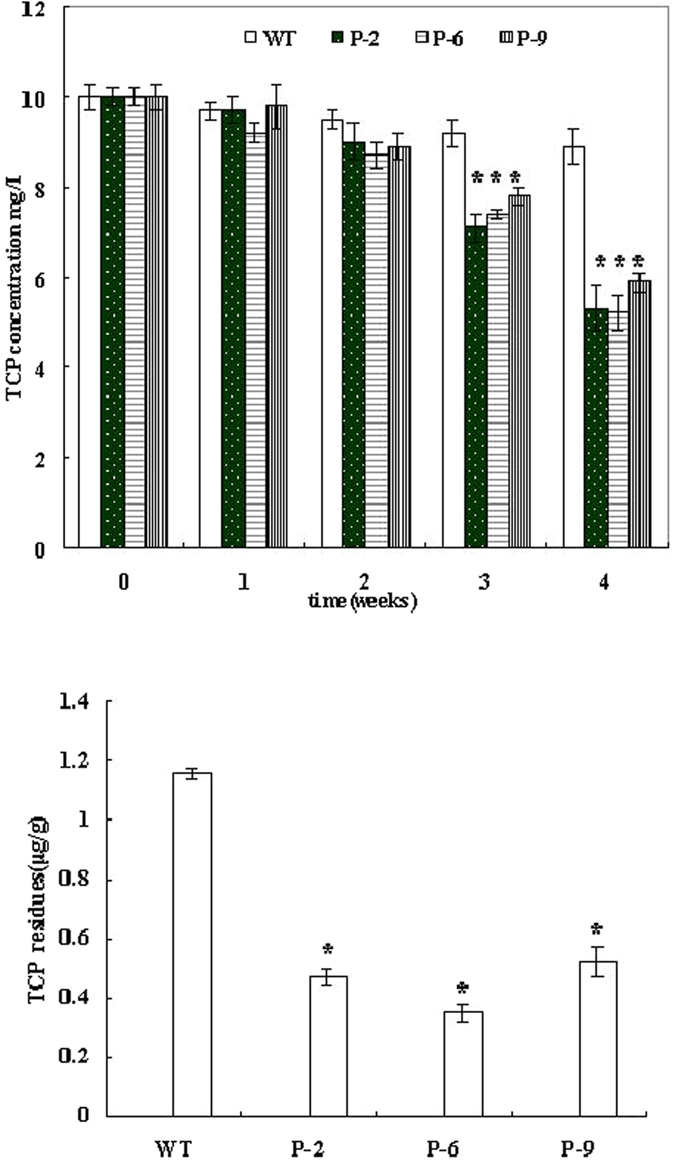
Removal of 2,4,6-TCP in the media by *AtPDIL1*-*2* transgenic plants. (**A**) 2,4,6-TCP degradation rates during plant incubation. 2,4,6-TCP degradation was calculated with the remaining TCP in the medium every week for a month after seedlings were grown in 1/2MS liquid media containing 10 mg/L 2,4,6-TCP. The natural loss of 2,4,6-TCP has been subtracted. (**B**) Comparison of residues of 2,4,6-TCP in *AtPDIL1*-*2* transgenic plants and WT treated with 10 mg/L of 2,4,6-TCP for a month. mAU, milli absorbance unit. *mean that the relative TCP residue in the culture medium is significantly different (p < 0.05) from that of WT plants (three different tests).

We previously found that plants can absorb 2,4,6-TCP from their growing environment through direct contact and that this compound accumulates *in*-*vivo* in a dose-dependent manner. Considering that AtPDIL1-2 can bind with TCP, we assumed that AtPDIL1-2 overexpressed in transgenic plants is responsible for TCP detoxification. We monitored the residual TCP in the medium for plants every week. The amount of 2,4,6-TCP in the media significantly decreased in the transgenic plants compared with the WT plants. The *AtPDIL1*-*2* transgenic plants P-6 generally showed about 2.5 times higher degradation rates than the WT plants after a month (Fig. 4A). We quantified the amounts of 2,4,6-TCP in plants. Results showed that the contents of 2,4,6-TCP in transgenic plants were 0.35–0.52 μg and 1.16 μg per gram fresh weight in the WT plants, respectively (Fig. 4B).

## Discussion

Trichlorophenols are persistent environmental pollutants. To search for valuable gene resources for the remediation and degradation of TCPs, we used gene chip assay to investigate the transcriptome of *Arabidopsis* in response to TCP stress. Among a set of responsive genes, we found that the PDI-like gene *AtPDIL1*-*2* was significantly upregulated by TCP stress (Supplementary Table 1 and Fig. 1). These results prompt us to investigate the role of AtPDIL1-2 in plants under TCP stress.

PDI proteins belong to an ancient protein family and are abundant in divergent organisms^27^. PDI mainly catalyzes the formation, isomerization, and reduction of disulfide bonds during protein folding through interaction with polypeptides. PDI can also act as a chaperone by binding with unfolded proteins noncovalently to prevent their aggregation^18^. Thus, PDI protein can be used to improve plant adaptation to different adversities. Overexpression of MTH1745, a stress-induced putative PDI from the thermophilic archaea *Methanothermobacter thermoautotrophicum*, could increase the stability of proteins and enhance Hg tolerance in rice^28^. Plants contain many PDI-like genes that differ in the length of polypeptides, the presence of signal peptides and ER retention signals, and the number and positions of thioredoxin and transmembrane domains^19^,^29^. Although the biochemical function of most plant PDIL proteins remains to be confirmed, biochemical studies showed that the major activity of PDIL is associated with the ER. The expression of six *AtPDI* genes increases in response to dithiothreitol and beta-mercaptoethanol, two reducing agents that can break disulfide bonds and inhibit their formation^21^. In the present study, we found that 2,4,6-TCP, even though is not a reducing agent, can also induce the expression of the PDI protein AtPDIL1-2. In mammals, Zhang *et al*. found that 2,4,6-TCP could induce ER stress by activating protein kinase-like ER kinase and inositol requiring enzyme/endonuclease 1α^9^. As the entry point into the secretory pathway, the ER is an important site containing an oxidizing environment for disulfide bond formation^30^. Considering that 2,4,6-TCP is highly lipophilic and AtPDIL1-2 has a typical ER retention signal at its C-terminal (Fig. 2A), we speculate that 2,4,6-TCP may trigger ER stress in *Arabidopsis* and activate AtPDIL1-2 expression to increase the protein-folding capacity in the ER.

Many studies have shown that PDI can serve as a binding protein for small molecules that contain a phenolic structure. The membrane-associated AtPDIL1-2 was also shown to bind with 2,4,6-TCP directly (Fig. 1D). The binding activity of 2,4,6-TCP to AtPDIL1-2 inhibited PDI-mediated isomerase activity (Fig. 1E). PDI covalently binding to peptides and proteins is essential for its effective catalysis of disulfide formation and isomerization. BPA exhibits inhibitory effects on PDI-mediated isomerase activity and disrupts various physiological functions by binding with PDI^26^. PDI can also serve as an intracellular binding protein for E2 and T3, two hormones that contain a phenolic structure^25^. Similar to other phenolic chemicals, 2,4,6-TCP may also exert inhibitory effects on AtPDIL1-2 during the catalysis of disulfide formation and isomerase activity through its binding activity with the PDI-like protein. The function of AtPDIL1-2 can be compensated by heterologous overexpression in *E. coli* and tobacco. The protein can enhance their 2,4,6-TCP tolerance by decreasing the concentration of the toxic compound in cells (Figs 1C and 4).

On PDI, two binding sites are available for thyroid hormone T3 and one binding site for endogenous hormones E2^25^. Binding of rat PDI with BPA on a single site inhibits its activity^26^. Given its structure, AtPDIL1-2 is a classical PDI containing four thioredoxin-like domains a-b-b′-a′ (Fig. 3A)^31^,^32^. To understand the structural principles for the binding of AtPDIL1-2 to 2,4,6-TCP, we screened the protein–ligand interaction using AutoDock, an effective tool for performing molecular docking to predict ligand–receptor interactions. This method has become an important tool in drug discovery^33^,^34^. The results of AutoDock running of AtPDIL1-2 to 2,4,6-TCP provided a suitable configuration for an accurate description of the protein-ligand binding site on the b-b′ domain (Fig. 2C). The formation of a hydrogen bond between the hydroxyl group of 2,4,6-TCP and His254 of AtPDIL1-2 is important for the binding interaction. The binding model is similar to human PDI’s E2-binding site^35^. Comparison of the sequences of the AtPDI b-b′ domain clearly showed that it is largely different at this region^21^, suggesting that not all AtPDI proteins display 2,4,6-TCP binding activity.

Used as a common chemical intermediate and a by-product of water chlorination and combustion, TCP is an environmental pollutant of concern in many countries. Plants were able to release extracellular peroxidase to oxidize phenolic xenobiotics *in vitro*^36^. However, given its hydroxyl group, TCP is easily absorbed by and accumulates readily in plants^37^. The high phytotoxicity and bioaccumulation ability of TCPs in organisms inspired us to develop a method to decrease TCP concentration in plant cells. We previously presented a system to degrade 2,4,6-TCP in plants based on sugar conjugation by overexpressing a UDP-glc-dependent glycosyltransferase and significantly enhanced the tolerance of transgenic plants to this pollutant^13^. In the present study, we designed a novel method to decrease TCP concentration in plants by binding with overexpressed AtPDIL1-2. The PDIL proteins can not only wrap up the TCP but also increase plant resistance to the toxin through its disulfide isomerase function. Our research on *AtPDIL1*-*2* transgenic tobacco implied that the constitutive expression of AtPDIL1-2 in plants is an effective method to remediate a TCP-contaminated environment.

## Materials and Methods

### Microarray experiments

Seeds of *A. thaliana* were surface sterilized for 20 min in 8% bleach, washed five times with sterile water, and then plated on half-strength Murashige and Skoog (1/2MS) medium containing 1% sucrose and 0.8% (w/v) agar, pH 5.8. Sterilized seeds were stratified at 4 °C for 2 days in darkness and then transferred to a climate chamber maintained under 16 h of light (120 μmol photons m^−2^ s^−1^) and 8 h of dark at 22 °C.

After germination for a week, the seedlings were transferred to fresh medium (with 0 and 50 μM 2,4,6-TCP) for 6 days. Three replications with 12 plants in each were prepared. The whole plants were harvested and immediately frozen in liquid nitrogen for RNA extraction. Total RNA from pooled tissue was isolated and then purified using Trizol reagent (Invitrogen, USA) in accordance with the manufacturer’s instructions. mRNA was extracted from the total RNA pools using Oligotex mRNA mini kit (Qiagen Inc., Valencia, CA, USA) and then reverse-transcribed to cDNA using SuperScript II RT (Invitrogen, Carlsbad, CA, USA) and T7-(dT)24 primer. Cy5- and Cy3-labeled cDNA probes were prepared and hybridized to *Arabidopsis* 2 oligo-microarrays (Agilent) for 17 h at 60 °C in a hybridization oven (Agilent). The hybridized slides were scanned using an Agilent DNA Microarray Scanner, and data points were extracted using Agilent Feature Extraction software (Agilent, Palo Alto, CA, USA)^15^.

### Expression of ATPDIL1-2 in *E. coli* and determination of PDI activity

Total RNA was reverse transcribed using an RNA PCR kit (Takara Bio, Inc., Japan) in accordance with the manufacturer’s instructions. The fragment of *ATPDIL1*-*2* was amplified with the forward primer PDILZ 5′-AGAGGGATCCATGGCGTTTAAGGG TTTC GCGTG-3′ and reverse primer PDILF 5′-AGAGAGCTCCTACAGCTCGTC CTTTGCGGCCGTT-3′. PCR was carried out for 30 cycles using Pyrobest DNA polymerase (Takara Bio, Inc.) as follows: denaturation at 94 °C for 30 s, annealing at 60 °C for 60 s, and extension at 72 °C for 120 s. After digestion with *Bam*HI and *Sac* I, the fragment of *ATPDIL1*-*2* was ligated with the pBluescript vector (Stratagene, La Jolla, CA). Following sequencing, the correct clone containing the full-length *ATPDIL1*-*2* cDNA was digested with *Bam*HI and *Sac* I, and then inserted into the histidine-tagged expression vector pET-32a. The resulting plasmid was transferred into *E. coli* BL21. To assay TCP resistance, the transformants were diluted to 10^3^ cells/μL, and 1 μL of cells was transferred to LB medium supplemented with 250 or 500 μM 2,4,6-TCP before incubation at 37 °C for 12 h.

ATPDIL1-2 expression was induced for 4 h by adding 1.0 mm IPTG after *E. coli* cells were grown at 37 °C for 6 h. The cells were harvested, lysed in a lysis buffer [50 mM Tris (pH 8.0) containing 300 mm NaCl, 10 mm imidazole, 1.0 mg/ml lysozyme, and 0.5% protease inhibitor cocktail] for 60 min at 4 °C, and then sonicated for 5 min. The cell lysate was centrifuged at 12,000 × *g* for 30 min, and the supernatant was loaded onto a nickel-chelate-nitrilotriacetic acid agarose column (https://www.qiagen.com). After elution with a buffer containing 250 mm imidazole and 0.1% sucrose monolaurate, the eluted fraction was dialyzed against 50 mM Tris-HCl buffer (pH 7.5) at 4 °C for 12 h. Protein concentration was measured using the protein assay kit (Shenggong, Shanghai, China).

The isomerase activity of the recombinant ATPDIL1-2 was assayed by the formation of native RNase, which was measured spectrophotometrically by monitoring the hydrolysis of the RNase substrate cCMP (reference) at 296 nm every 5 min for 60 min^38^. Reduced, denatured RNase (10 μM) was added to 3.5 μM recombinant ATPDIL1-2 in a buffer containing 4.5 mM cCMP, 1 mM glutathione, 0.2 mM glutathione disulfide, 2 mM EDTA, and 100 mM Tris-HCl, pH 8. Reduced RNase A was prepared by overnight incubation RNase A (5 mg) in 1 mL of denaturing buffer (140 mM DTT, 2 mM EDTA, 6 M guanidinum chloride, 100 mM Tris-HCl, pH 8).

### ATPDIL1-2 and TCP binding assay

The purified recombinant PDI (3.5 μM) was incubated with 0.05 mM 2,4,6-TCP (dissolved in methanol at 50 mM) in 0.5 mL of binding buffer [50 mm Tris-HCl buffer (pH 7.5) containing 150 mm NaCl] for 0–3 h at 4 °C^26^. Reactions were stopped by adding 80% methanol. The residual TCP was determined using HPLC in an Agilent 1100 system (Wilmington, DE, USA) on a 4.6 mm × 150 mm Athena C18 column (Anpel, Shanghai, China) in 70% aqueous methanol at a flow rate of 800 μL/min with UV detection at 210 nm^15^.

### Ligand docking and binding site analysis of TCP and ATPDIL1-2 with AutoDock

The structure of ATPDIL1-2 was modeled by SWISS-MODEL based on the structures of typical PDI ERp57 (PDB 3f8u)^39^. Automated docking was used to locate the appropriate binding orientations and conformations of 2,4,6-TCP in the PDI binding pocket^40^. To perform the task, genetic algorithm routine implemented in the program AutoDock 4.2.6 (http://autodock.scripps.edu) was employed. All water molecules were removed from the ATPDIL1-2. Polar hydrogens and partial Kollman charges were assigned, and then atomic solvation parameters and fragmental volumes were allotted to the protein using AutoDock Tools (ADT). The program AutoGrid was used to generate the grid maps that represent the intact ligand in the actual docking target site. A grid box was used to prepare a grid map with a size of 60 × 60 × 60 Å *xyz* points. The grid center was designated at dimensions (*x, y, z*): 16.828, 78.553, 63.379 with a spacing of 0.375 Å. The standard docking protocol for rigid and flexible ligand docking consisted of 10 independent runs per ligand. After docking, 10 models were calculated, and the one with the lowest binding energy was chosen for further energy minimization. In AutoDock, the overall docking energy of a given ligand molecule is expressed as the sum of intermolecular interaction energies, including van der Waals attractive and repulsive energies, H-bond interaction energy, coulombic electrostatic energy, and the internal steric energy of the ligand.

### Construction of plant expression vector and plant transformation

The fragment of *ATPDIL1*-*2* was digested with *Bam*HI and *Sac*I, and then cloned into the plant expression vector pCAMBIA-1301 under the control of the cauliflower mosaic virus 35S promoter. The final constructs were introduced into *Agrobacterium tumefaciens* LBA4404 (Clontech, Palo Alto, CA) by electroporation and then transferred into tobacco (*Nicotiana tabacum* cv *Xanthi*) through the leaf disc method^41^. Shoots were rooted on a medium containing 30 mg/L hygromycin, transferred into soil, and then grown in a greenhouse.

### Transgenic plant selection and assay for TCP resistance

RT-PCR was performed to confirm the transcription of *ATPDIL1*-*2*. First-strand cDNA of T2 generations was synthesized using 5 μg of total RNA with the Reverse Transcription System (Promega, Madison, WI, USA). The tobacco *Actin* gene, used as an internal standard to normalize the amount of cDNA, was amplified using primers TactZ (5′-CAATGAACTTCGTGTGGCTCC-3′) and TactF (5′-CGGAATCTCT CAG CACCAATG-3′)^42^. A specific fragment of about 267 bp from the 3′ domain of the *ATPDIL1*-*2* gene was amplified with the primer 5′-ACGAAGTGGCTTTGTCATTC-3′ and 5′-TTTCCTCGGTCTTCTTAGGT-3′ from transgenic plants using the same amount of cDNA. The PCR products were separated on 2% agarose gel and then quantified using a Model Gel Doc 1000 (Bio-Rad, USA). The expression pattern of the *ATPDIL1*-*2* gene was evaluated with a Shine Tech Gel Analyzer (Shanghai Shine Science of Technology Co., Ltd., China). The same results were obtained for three independent experiments.

Seeds of T2 generations of transgenic tobacco were surface sterilized with bleaching powder (5%, *w*/*v*) for 20 min, washed three times with sterile water, and then sown in 1/2MS medium containing 10 mg/L 2,4,6-TCP. The plates were incubated at 4 °C for 2 days in darkness to synchronize germination. The seeds were incubated at 25 °C for 10 days in growth chambers under a constant illumination with cool-white fluorescent lamps for 16 h and a dark period of 8 h daily. Finally, the root length and fresh weight of approximately 10 seedlings were measured.

To observe root development, 4-day-old seedlings were transferred to a fresh tube supplemented with 2,4,6-TCP at a concentration of 10 mg/L. The tube was placed in the greenhouse at 22–25 °C for 4 weeks. For assay of plants grown in soil, 4-day-old seedlings were transferred to a pot containing synthetic soil and a mixture of peat/vermiculite/perlite (1:8:1, *v*/*v*/*v*). After the seedlings were grown for 3 days, the pot was soaked with 20 mg/L TCP solution for 5 min. Two weeks later, the seedlings were sprayed twice with the same concentration of TCP solution with a 7-day interval.

### Phytoremediation of TCP

To determine whether the transgenic plants can remediate TCP, the residual TCP in the medium was determined by HPLC^15^. A triangular flask containing 20 mL of 1/2MS liquid medium with 10 mg/L TCP was used to grow tobacco seedlings. The seeds of transgenic or non-transgenic tobacco were germinated, transferred into the triangular flask, and then placed on a small shaker at a rotation speed of 50 rpm at 25 °C for 4 weeks. To reduce TCP volatilization from the system, the tubes were enclosed with plastic material and then tied with a strong rubber band. The culture medium was extracted with a syringe and then added with methanol at a ratio of 1:1 every week. The medium was centrifuged (12,000 rpm, 10 min), and the supernatant was filtered through a 0.22 μm filter. The accumulation of 2,4,6-TCP *in*-*vivo* of tobacco seedlings was also analyzed by HPLC. A month later, the whole plant (including roots, stem, and leaves) was harvested and homogenized with a pestle in a pre-cooled mortar in 0.5 mL of methanol. To reduce the contamination of the residual TCP in the medium, the roots were washed three times with 30% methanol and blotted with paper to remove any excess material before homogenization. The homogenized tissues were centrifuged (12,000 rpm, 5 min), and the supernatant was filtered through a 0.22 μm filter. The 2,4,6-TCP standard was diluted and analyzed by HPLC, and the quantification of TCP was related to the peak area. The recovery of 2,4,6-TCP was determined by comparing the peak area ratios in the extracted samples to those in standard solutions.

### Statistical analysis

All experiments were carried out using three replicates. The results between treatments were analyzed by one-way analysis of variance (ANOVA). Statistical significance was evaluated by the F-test at the 5% level (95% confidence level).

## Additional Information

**How to cite this article:** Peng, R.-H. *et al*. Disulfide isomerase-like protein AtPDIL1-2 is a good candidate for trichlorophenol phytodetoxification. *Sci. Rep.* **7**, 40130; doi: 10.1038/srep40130 (2017).

**Publisher's note:** Springer Nature remains neutral with regard to jurisdictional claims in published maps and institutional affiliations.

## Supplementary Material

Supplementary Materials
