# Supplementary material for: Disulfide isomerase-like protein AtPDIL1–2 is a good candidate for trichlorophenol phytodetoxification
Source: Sci Rep. 2017 Jan 6;7:40130. doi: 10.1038/srep40130 (PMC5216352; doi:10.1038/srep40130)
Supplement: Supplementary Materials [file srep40130-s1.doc]

**Disulfide isomerase-like protein AtPDIL1-2 is a good candidate for trichlorophenol phytodetoxification**

Ri-He Peng^1^, Jin Qiu^1^, Yong-Sheng Tian, Jian-jie Gao, Hong-juan Han, Xiao-Yan Fu, Bo Zhu, Jing Xu, Bo Wang, Zhen-jun Li, Li-juan Wang, Quan-Hong Yao *

Agro-Biotechnology Research Institute, Shanghai Academy of Agricultural Sciences; Shanghai Key Laboratory of Agricultural Genetics and Breeding.

2901 Beidi Rd., Shanghai, People’s Republic of China

***Corresponding author:**

Prof. Quanhong Yao

Director and Professor

Shanghai Key Laboratory of Agricultural Genetics and Breeding

Agro-Biotechnology Research Center

Shanghai Academy of Agricultural Sciences

2901 Beidi Rd, Shanghai. P. R. China

Tel: 086-021-62203180

Fax: 086-021-62203180

Email address: **yao.quanhong65@yahoo.com**

^1^These authors contributed equally to this work.

**Supplemental materials**

Supplementary table S1

The change of different PDIL genes expression in response to 50 μM 2,4,6-TCP (Arabidopsis seedlings exposed to 50 μM 2,4,6-TCP for 6 days) in the Agilent Arabidopsis microarrays. Microarray data for normalization fold expression are calculated from three biologically independent experiments.

The change of different PDIL genes expression in response to 50 μM 2,4,6-TCP.

| Accession No. of gene | PDIL designation | Fold change |
| --- | --- | --- |
| At1g21750 | AtPDIL1-1 | 1.46 |
| At1g77510 | AtPDIL1-2 | 2.39 |
| At3g54960 | AtPDIL1-3 | 1.67 |
| At5g60640 | AtPDIL1-4 | 1.46 |
| At1g52260 | AtPDIL1-5 | -1.73 |
| At3g16110 | AtPDIL1-6 | -1.03 |
| At2g47470 | AtPDIL2-1 | -1.23 |
| At1g04980 | AtPDIL2-2 | 1.57 |
| At2g32920 | AtPDIL2-3 | 1.16 |
| At1g07960 | AtPDIL5-1 | -1.02 |
| At1g35620 | AtPDIL5-2 | -1.01 |
| At3g20560 | AtPDIL5-3 | -1.06 |
| At4g27080 | AtPDIL5-4 | 1.08 |
| At1g15020 | AtQSOX1 | -1.05 |
| At2g01270 | AtQSOX2 | -1.39 |
| At4g04610 | AtAPR1 | 1.08 |
| At1g62180 | AtAPR2 | -1.15 |
| At4g21990 | AtAPR3 | 1.04 |
| At1g34780 | AtAPRL4 | 1.09 |
| At3g03860 | AtAPRL5 | 1.29 |
| At4g08930 | AtAPRL6 | 1.29 |
| At5g18120 | AtAPRL7 | 1.17 |

Fold change indicates the mRNA ratios of 2,4,6-TCP-treated to untreated plants. “-” indicated gene down- regulated.

Supplementary Figure S1

0 10 20 30

*AtPDIL1-1*
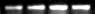
 *AtPDIL1-2*
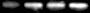
 ACTIN2
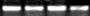


Fig. S1. RT-PCR analysis of *AtPDIL1-1* and *AtPDIL1-2* after 2,4,6-TCP treatment by microarray analysis. Seedlings were subject to 2,4,6-TCP treatment (0, 10, 20, and 30μM). The expression of *ACTIN2* gene was used to adjust cDNA concentrations.
